# [^13^C_6_,D_8_]2-deoxyglucose phosphorylation by hexokinase shows selectivity for the β-anomer

**DOI:** 10.1038/s41598-019-56063-0

**Published:** 2019-12-23

**Authors:** Gal Sapir, Talia Harris, Sivaranjan Uppala, Atara Nardi-Schreiber, Jacob Sosna, J. Moshe Gomori, Rachel Katz-Brull

**Affiliations:** Department of Radiology, Hadassah Medical Center, Hebrew University of Jerusalem, The Faculty of Medicine, Jerusalem, Israel

**Keywords:** Biochemical assays, Deoxy sugars, Diagnostic markers

## Abstract

A non-radioactive 2-deoxyglucose (2DG) analog has been developed here for hyperpolarized magnetic resonance investigations. The analog, [^13^C_6_,D_8_]2DG, showed 13% polarization in solution (27,000-fold signal enhancement at the C_1_ site), following a dissolution-DNP hyperpolarization process. The phosphorylation of this analog by yeast hexokinase (yHK) was monitored in real-time with a temporal resolution of 1 s. We show that yHK selectively utilizes the β anomer of the 2DG analog, thus revealing a surprising anomeric specificity of this reaction. Such anomeric selectivity was not observed for the reaction of yHK or bacterial glucokinase with a hyperpolarized glucose analog. yHK is highly similar to the human HK-2, which is overexpressed in malignancy. Thus, the current finding may shed a new light on a fundamental enzyme activity which is utilized in the most widespread molecular imaging technology for cancer detection – positron-emission tomography with ^18^F-2DG.

## Introduction

The hexokinase (HK) family of enzymes carry out the first step in glycolysis and therefore are of key importance in cellular metabolism^1^. HKs play an important role in malignancy: one of the earliest observations in malignant transformation is an increased glycolytic flux^2^, which is largely attributed to changes in the activity of HKs. For example, in rapidly growing tumor cells, HK expression is markedly elevated and most of the enzyme is localized to the mitochondrial membrane^3,4^, which provides HK with increased ATP availability resulting in increased activity^5^.

HK isoenzymes vary in function and in organ expression. In health, HK-4 (glucokinase, GK) is important in glucose sensing in the pancreas and in the liver^6^ while HK-1 and HK-2 mainly function in other tissues such as muscle and brain. Increased or decreased HK expression has been found to correlate with the clinical outcome in a number of disease states^7–20^. Elevated HK-2 expression is correlated with higher histological grade in hepatocellular carcinoma (HCC)^7^ as during the process of HCC tumorigenesis, normal GK expression is silenced, and HK-1 and HK-2 are overexpressed^8,9^. Since the affinity of HK-1 and HK-2 for glucose is higher than that of GK, their expression results in increased glucose utilization which supports HCC tumorigenesis. HK-2 expression was shown to be correlated with the survival of patients with HCC, gastric and colorectal cancer, and WHO grade IV glioblastoma^10–13,20^. Aggressive types of cancer including pancreatic ductal adenocarcinoma^14^, renal cell carcinoma^15^, medulloblastoma^16^, and mouse models of lung and breast cancer^17^ are characterized by high levels of HK-2. Other non-malignant diseases with increased HK-2 expression in the liver include fatty liver disease^18^, and hepatitis C virus infection^19^.

[^18^F]Fluoro-2-deoxy-D-glucose (FDG) is the most widely used tracer in positron emission tomography (PET) for detecting, staging, and monitoring of various malignancies^21^. It was previously suggested that the increased HK-2 activity forms the basis for the utility of FDG-PET imaging of malignant tumors^5^. Nuclear magnetic resonance (NMR) has been used for the study of glucose and 2-deoxyglucose (2DG) metabolism^22–25^. In ^13^C-NMR, as opposed to PET, the chemical evolution of 2DG to 2DG-6-phosphate (2DG6P) can be discerned. This property could be useful for differentiating the effects of glucose transporters expression from those of HK expression *in vivo*. For the above reasons, glucose and 2DG imaging has been a desired target in magnetic resonance imaging, which does not involve ionizing radiation. MR investigations of glucose and 2DG have been carried out using the gluco-CEST approach^22,26,27^, thermal equilibrium ^13^C-NMR with carbon-13 labeled substrates^28^, and deuterium metabolic imaging (DMI)^29^.

The introduction of the dissolution-dynamic nuclear polarization (dDNP) technique has allowed more than a 10,000-fold increase in the observed ^13^C-NMR signal in solution^30^, and thus enabled research of fast biochemical conversion processes on time scales of seconds, not possible with thermal equilibrium ^13^C-NMR. dDNP was extensively used to study *in vivo* enzymatic conversion processes in animal models of health and disease^31^ and recently in humans^32–37^. dDNP-NMR was also used in *in vitro* studies such as kinetics of enzymatic reactions^38–41^, reactive oxygen species production^42^, protein-substrate interactions^43^, protein folding^44^, and diffusion in solution^45^. With regards to glucose metabolism, hyperpolarized [^13^C_6_,D_7_]glucose was used to demonstrate HK-mediated conversion to G6P, and G6P inhibition of this reaction^38^. Hyperpolarized [^13^C_6_,D_7_]glucose was also used to study metabolism in cell cultures^46^ and its distribution in the body was demonstrated in real-time by hyperpolarized MRI^47^. Real-time monitoring of hyperpolarized [^13^C_6_,D_7_]glucose metabolism *in vivo*, in a lymphoma model in mice, showed lactate production in the tumor, but not in surrounding tissues^48^, and an injection of hyperpolarized [^13^C_6_,D_7_]glucose to the normal mouse brain was followed by observation of lactate and pyruvate by ^13^C MRS in sub-second temporal resolution^49^. These latter studies demonstrate the ability to monitor the metabolic conversions of [^13^C_6_,D_7_]glucose in a hyperpolarized state despite the fast metabolism and despite the relatively shorter life time of the hyperpolarized state^47^ compared to other dDNP probes. However, the fast metabolism and conversion to the freely diffusing [^13^C]CO_2_ also hinders the ability to image this powerful agent with hyperpolarized MRI. For this reason, the PET examination utilizes 2DG – a glucose derivative that undergoes phosphorylation only, whereas this phosphorylated form remains intracellular.

To develop an MRI parallel of the FDG-PET examination we have previously designed a 2DG analog with similar isotopic-labeling strategy as that used for hyperpolarized glucose studies, *i.e*. a uniform ^13^C label and deuteration of all SP^3^ carbons ([^13^C_6_,D_8_]2DG)^50^. Here we have studied the HK activity on this analog in a hyperpolarized state. A fast acting, readily available HK from the yeast *Saccharomyces cerevisiae* (yHK) was used here as a model for mammalian HKs due to its high similarity^51–53^ (further information is provided in the Discussion and Supplementary Information S3). As a control, we used the activity of this enzyme on hyperpolarized [^13^C_6_,D_7_]glucose. Studies with bacterial GK (bGK) which bears no similarity to the human enzymes and which is not expected to react with 2DG^54,55^ were also performed and served to highlight traits in the spectral consequences of enzymatic activity on [^13^C_6_,D_8_]2DG and [^13^C_6_,D_7_]glucose.

Using a comprehensive kinetic analysis and decomposition of the complex and overlapping [^13^C_6_,D_8_]2DG and [^13^C_6_,D_8_]2DG6P signals we found a differential activity of the yHK on the α and β anomers of [^13^C_6_,D_8_]2DG as opposed to a non-differential activity on [^13^C_6_,D_7_]glucose anomers. The high temporal resolution (seconds) enabled by the hyperpolarized MR technology was key in the ability to perform this study and reach this conclusion. The importance of this finding for understanding HK activities in cancer warrants further investigation and is important for the development of such a potential tracer for hyperpolarized MRI.

## Results

### [^13^C_6_,D_8_]2DG is visible in a hyperpolarized state

**Figure 1:**
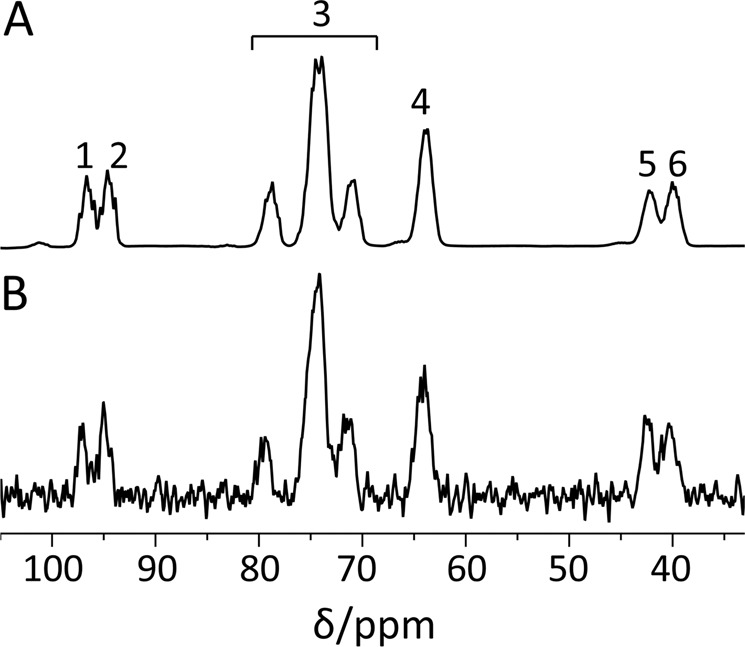
^13^C-NMR spectra of [^13^C_6_,D_8_]2DG in a hyperpolarized state and at thermal equilibrium. (**A**) A ^13^C spectrum of hyperpolarized [^13^C_6_,D_8_]2DG recorded with a single scan using a 10° flip angle. (**B**) A spectrum of the same sample of [^13^C_6_,D_8_]2DG at thermal equilibrium acquired over 84 min using 64 excitations, 80 s repetition time, and the same flip angle (10°). The spectral processing for both spectra consisted of 10 Hz exponential line broadening and zero-filling from 8,192 to 16,834 points. Signal assignment: 1) C_1_β; 2) C_1_α, 3) C_3_, C_4_, and C_5_; 4) C_6_; 5) C_2_β; 6) C_2_α. The chemical shift scale was referenced to C_6_ of 2DG (signal 4) at 64.0 ppm^25^.

Following 1.8 to 2.3 h of polarization in the dDNP polarizer (formulation and polarization described in the Methods), [^13^C_6_,D_8_]2DG was observed in a hyperpolarized state in solution as described in Table 1. Typical ^13^C-NMR spectra of [^13^C_6_,D_8_]2DG in a hyperpolarized state and at thermal equilibrium are shown in Fig. 1.

**Table 1:** Enhancement factors and polarization percent determined for ^13^C sites of hyperpolarized [^13^C_6_,D_8_]2DG^*^.

^13^C site in [^13^C_6_,D_8_]2DG	Enhancement factor in solution at 5.8 T	Polarization percent in solution
C_1_	27,395 ± 10,261	13.2 ± 4.8
C_6_	22,234 ± 7,539	11.1 ± 3.8

^*^The measurements were done in the same buffer used for the enzymatic experiments (described below). A total of three measurements are reported. Two measurements were conducted at 40 °C (one with bGK and one without an enzyme), and one measurement was conducted at room temperature (without an enzyme).

### The phosphorylation of [^13^C_6_,D_8_]2DG is detectable in real-time by hyperpolarized NMR in the reaction with yHK

**Figure 2:**
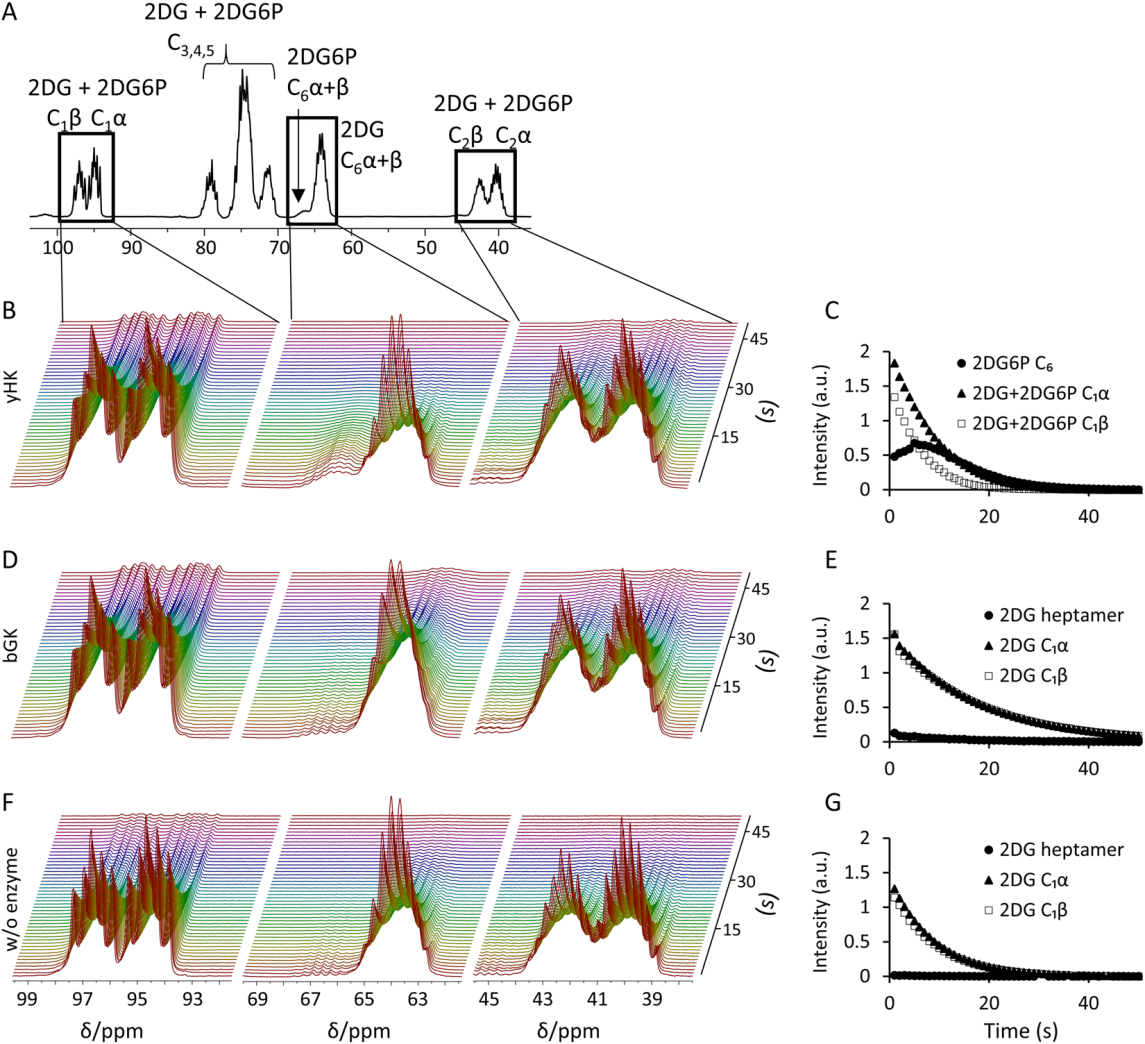
Phosphorylation reactions of [^13^C_6_,D_8_]2DG by yHK and bGK. (**A**) A ^13^C spectrum showing all of the signals of hyperpolarized [^13^C_6_,D_8_]2DG and [^13^C_6_,D_8_]2DG6P during the reaction with yHK. (**B**) Consecutive ^13^C spectra of a typical experiment with yHK. The chemical shift regions of the C_1_, C_6_, and C_2_ signals of both [^13^C_6_,D_8_]2DG and [^13^C_6_,D_8_]2DG6P are presented. (**C**) The time course of the integrated signal intensities for the experiment shown in B for the signals centered at 66.59 (2DG6P C_6_), 94.69 (C_1_α), and 96.69 (C_1_β) ppm. (**D**) Consecutive ^13^C spectra of a typical experiment with bGK. The same chemical shift regions as in B are presented. (**E**) The time course of the integrated signal intensities for the experiment shown in D for the same chemical shift regions presented in C. (**F**) Consecutive ^13^C spectra of a typical experiment without an enzyme. The same chemical shift regions as in B are presented. (**G**) The time course of the integrated signal intensities for the experiment shown in F for the same chemical shift regions presented in C. All spectra were acquired over 50 s after the addition of hyperpolarized [^13^C_6_,D_8_]2DG, with a repetition time of 1 s and a 10° flip angle. yHK – yeast hexokinase, bGK - bacterial glucokinase, w/o - without, Intensity - integrated signal intensity, a.u. - arbitrary units.

Next, we investigated the ability to follow the phosphorylation of [^13^C_6_,D_8_]2DG with yHK and with bGK. To this end, hyperpolarized [^13^C_6_,D_8_]2DG was directly injected to an NMR tube containing the enzymatic reaction buffer solution (Methods) and the yHK or bGK enzyme. In the reactions with yHK, hyperpolarized [^13^C_6_,D_8_]2DG6P was observed immediately – already in the first spectrum acquired after the arrival of the hyperpolarized medium into the NMR tube (within 1 s), as can be seen in Fig. 2A,B (middle panel, signal at 66.18 ppm), and 2C. However, the conversion of [^13^C_6_,D_8_]2DG to [^13^C_6_,D_8_]2DG6P was not observed in the reaction with bGK (Fig. 2D, middle panel). The low intensity multiplet observed at a chemical shift close to that of the C_6_ position of [^13^C_6_,D_8_]2DG6P is likely due to the heptamer form of this compound, as previously reported for glucose^56,57^. These signals also appear in the experiment carried out without an enzyme (Fig. 2F, middle panel). As the lack of reaction of bGK on 2DG observed here was in agreement with prior publications^54,55^, this experiment was carried out only twice.

Interestingly, in the reaction with yHK, the signals of the β anomer appeared to decay faster compared to the α anomer, with an apparent T_1_ that is about 31% shorter for the C_1_ position and 29% for the C_2_ position (Table 2). No significant differences were observed in the experiments carried out without an enzyme (Supplementary Information S1) or with bGK (Table 2).

**Table 2:** Longitudinal relaxation times of ^13^C sites* in [^13^C_6_,D_8_]2DG and [^13^C_6_,D_7_]glucose during phosphorylation reactions.

Compound & Enzyme	Apparent T_1_ of C_1_β (s)	Apparent T_1_ of C_1_α (s)	T_1_ of C_6_P (s)	T_1_ of C_6_ (s)	Apparent T_1_ of C_2_β (s)	Apparent T_1_ of C_2_α (s)
[^13^C_6_,D_8_]2DG & yHK^a, #^	8.8 ± 0.3	12.8 ± 1.1	7.2 ± 0.6	9.7 ± 0.2	5.9 ± 0.3	8.4 ± 0.8
[^13^C_6_,D_8_]2DG & bGK^b^ **	22.3 ± 2.1	22.0 ± 1.7	Not detected	12.6 ± 3.3	15.3 ± 0.6	15.1 ± 0.8
[^13^C_6_,D_7_]glucose & yHK^c^	10.3 ± 0.5	10.0 ± 0.4	6.4 ± 0.3	7.9 ± 0.1	Not resolved	Not resolved
[^13^C_6_,D_7_]glucose & bGK^c^ **	14.0 ± 0.8	14.8 ± 1.4	9.8 ± 1.6	13.0 ± 1.9	Not resolved	Not resolved

Values are presented as mean ± standard deviation.

^#^Differences in the apparent T_1_ between the β and α anomers for the C_1_ and C_2_ positions were 31% (p = 0.00004) and 29% (p = 0.0002), respectively. p-values were calculated using a paired t-test.

*Only sites that could be spectrally resolved were analyzed. The apparent T_1_ calculation (Methods) for the C_1_ and C_2_ sites was done without taking into consideration the reaction kinetics as the substrate and product signals were not resolved for this site. For the C_6_ position, a full kinetic analysis was performed and the reaction kinetic rate constant are provided in Supplementary Information S2.

**Experiments with bGK were performed at 40 °C. Experiments with yHK were performed at room temperature (*ca*. 21 °C). (a) n = 5; (b) n = 2 (no reaction); (c) n = 4.

C_6_P, C_6_ position of the phosphorylated product; C_6_, C_6_ position of the substrate.

### Quick [^13^C_6_,D_7_]glucose phosphorylation by both yHK and bGK is detectable in real-time in a hyperpolarized state

**Figure 3:**
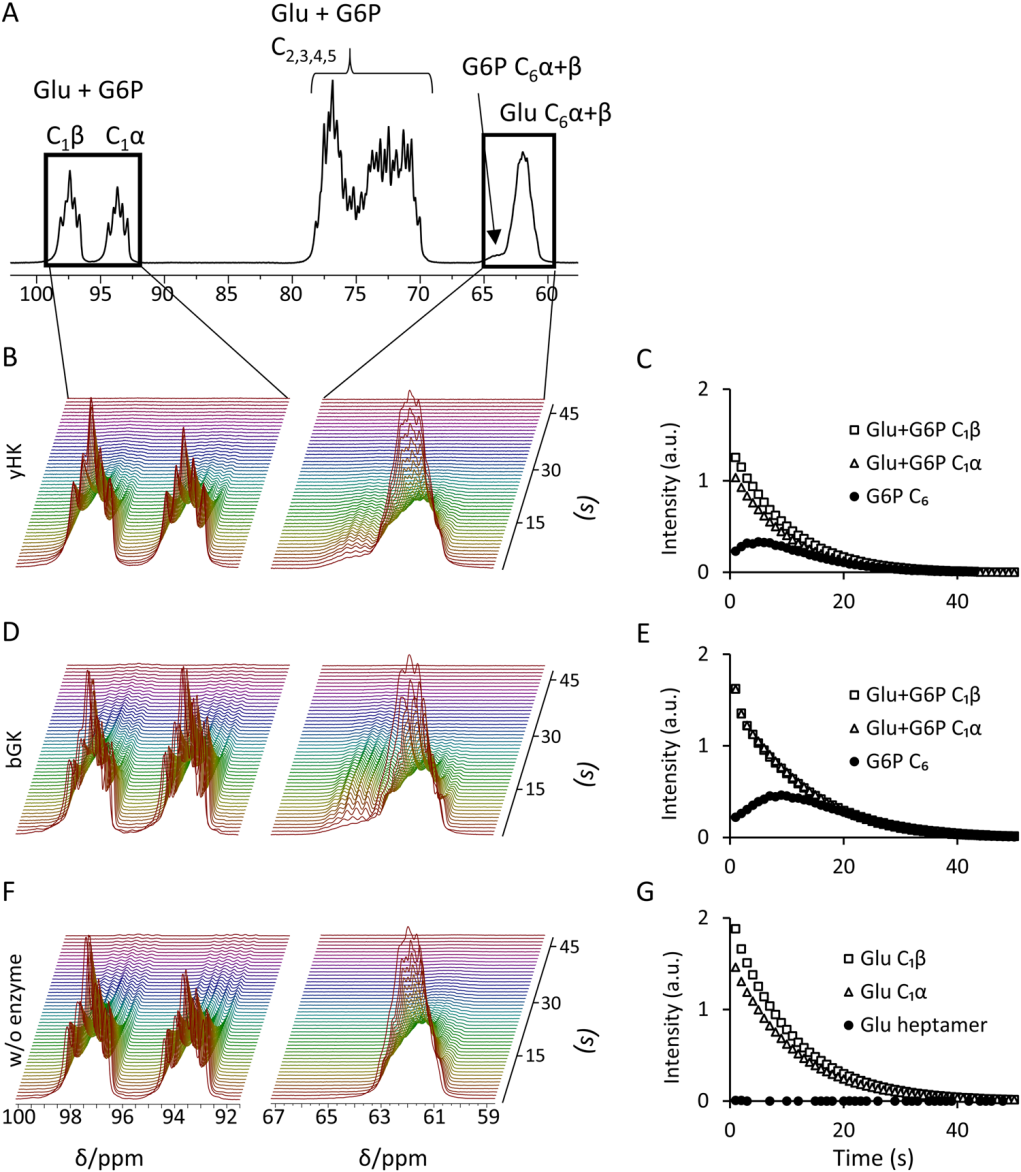
Phosphorylation reactions of [^13^C_6_, D_7_]glucose by yHK and bGK. (**A**) A ^13^C spectrum showing all of the signals of hyperpolarized [^13^C_6_,D_7_]glucose and [^13^C_6_,D_7_]G6P during the reaction with yHK. The chemical shift was referenced to C_1_β at 97.4 ppm^77^. (**B**) Consecutive ^13^C spectra of a typical experiment with yHK. The chemical shift regions of the C_1_ and C_6_ signals of both [^13^C_6_,D_7_]glucose and [^13^C_6_,D_7_]G6P are presented. (**C**) The time course of the integrated signal intensities for the experiment shown in B. (**D**) Consecutive ^13^C spectra of a typical experiment with bGK. The chemical shift regions of the C_1_ and C_6_ signals of both [^13^C_6_,D_7_]glucose and [^13^C_6_,D_7_]G6P are presented. (**E**) The time course of the integrated signal intensities for the experiment shown in D. (**F**) Consecutive ^13^C spectra of a typical experiment without an enzyme. The chemical shift regions of the C_1_ and C_6_ signals of both [^13^C_6_,D_7_]glucose and [^13^C_6_,D_7_]G6P are presented. (**G**) The time course of the integrated signal intensities for the experiment shown in F. The spectra were collected over 50 s after the addition of hyperpolarized [^13^C_6_,D_7_]glucose, with a repetition time of 1 s and a 10° flip angle. yHK – yeast hexokinase, b GK - bacterial glucokinase, w/o - without, Intensity - integrated signal intensity, a.u. - arbitrary units.

As a control for the experiments with [^13^C_6_,D_8_]2DG, we have tested the same reactions with hyperpolarized [^13^C_6_,D_7_]glucose. In these experiments, in the presence of both yHK (Fig. 3B) or bGK (Fig. 3D) the C_6_ signal of [^13^C_6_,D_7_]G6P builds up immediately and then decays, indicating the quick production of hyperpolarized [^13^C_6_,D_7_]G6P. As opposed to the reactions with [^13^C_6_,D_8_]2DG, [^13^C_6_,D_7_]glucose reacted also with bGK. As expected, in the experiment without an enzyme, the signal of [^13^C_6_,D_7_]G6P was not observed, although some experiments showed a faint signal at a nearby chemical shift – attributed to the heptamer form of [^13^C_6_, D_7_]glucose^56,57^. As opposed to the reaction of [^13^C_6_,D_8_]2DG with yHK, in both reactions with [^13^C_6_,D_7_]glucose the decay rate of the C_1_α and C_1_β signals appeared similar and these signals were characterized by similar apparent T_1_s (Table 2).

### Further analyses of the reactions with [^13^C_6_,D_8_]2DG and [^13^C_6_,D_7_]glucose

Table 2 shows the apparent T_1_ relaxation times that were determined for the signals of [^13^C_6_,D_8_]2DG and [^13^C_6_,D_7_]glucose in the presence of yHK, for those signals that could be spectrally resolved. In the reactions of [^13^C_6_,D_8_]2DG, the apparent T_1_s of the C_1_ and C_2_ carbons of the two anomers of the combined [^13^C_6_,D_8_]2DG and [^13^C_6_,D_8_]2DG6P signals were found to be different with the T_1_ of the β anomer signals being about 30% shorter than that of the α anomer in both positions. This effect was not observed for the reaction of [^13^C_6_,D_8_]2DG with bGK (where no phosphorylation occurred) and for the reactions of [^13^C_6_,D_7_]glucose with yHK or with bGK. We note that the T_1_ of the C_1_ position of both anomers in both [^13^C_6_,D_7_]glucose and [^13^C_6_,D_8_]2DG is longer at 40 °C compared to room temperature, with this effect being more pronounced for [^13^C_6_,D_8_]2DG and apparent also for its C_2_ position (Supplementary Information S1).

The C_6_ signal in these experiments was useful for characterizing the reaction rate constants as the substrate and the product signals were well resolved in this site. Therefore, the time course of the C_6_ signals of the substrate and product were fitted to a kinetic model which considers the metabolic reaction as a first-order reaction^39^. The metabolic rate constants that were obtained are provided in Supplementary Table S2.

### The reaction of [^13^C_6_,D_8_]2DG with yHK reveals a decrease in the β to α anomeric ratio

**Figure 4:**
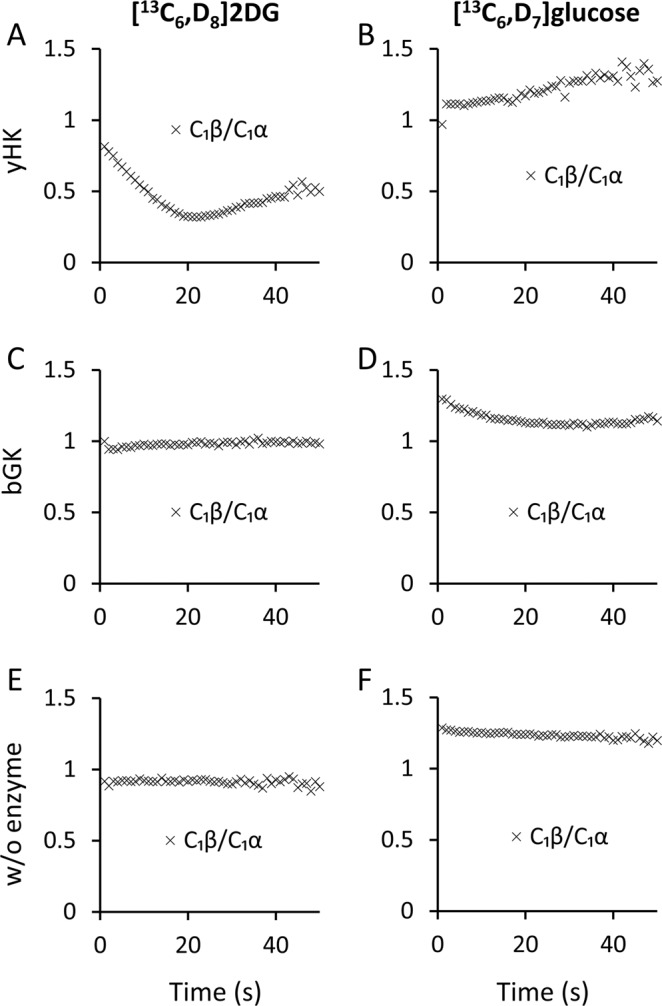
Anomeric ratios over time in [^13^C_6_,D_8_]2DG and [^13^C_6_,D_7_]glucose reactions. The plots show the time course of the β/α ratio of the integrated signal intensities of the C_1_ positions in typical experiments. (**A,B**) Reactions of [^13^C_6_,D_8_]2DG and [^13^C_6_,D_7_]glucose, respectively, with yHK. (**C,D**) Reactions of [^13^C_6_,D_8_]2DG and [^13^C_6_,D_7_]glucose, respectively, with bGK, (no reaction for the former). (**E,F**) Experiments with [^13^C_6_,D_8_]2DG and [^13^C_6_,D_7_]glucose, respectively, without an enzyme. yHK – yeast hexokinase, bGK - bacterial glucokinase, w/o - without.

Figure 4 shows the change in the ratio of anomers over the course of the reactions of [^13^C_6_,D_8_]2DG and [^13^C_6_,D_7_]glucose with yHK and bGK. In addition to the fast decay of C_1_β of [^13^C_6_,D_8_]2DG, this experiment also revealed different reaction kinetics for the two anomers present in the reaction medium. In Fig. 4A, it can be seen that the β/α ratio of the C_1_ position is decreasing during the first 20 s and then increasing. We wished to determine if this behavior is unique to [^13^C_6_,D_8_]2DG by inspecting the same parameter in the experiments with [^13^C_6_,D_7_]glucose. As shown in Fig. 4B, the reaction of [^13^C_6_,D_7_]glucose with yHK did not show this behavior, rather - a slightly increasing β/α C_1_ ratio can be seen during the same reaction time.

To further investigate this behavior, we analyzed the reactions of both substrates with bGK in the same way. Figure 4C,D show this analysis for [^13^C_6_,D_8_]2DG and [^13^C_6_,D_7_]glucose, resulting in a relatively constant ratio throughout the observation window. The experiments without enzymes (Fig. 4E,F for [^13^C_6_,D_8_]2DG and [^13^C_6_,D_7_]glucose, respectively) also showed a C_1_ β/α ratio that is relatively constant, all in contrast to the reaction of [^13^C_6_,D_8_]2DG with yHK.

### Quantification of the anomer specific substrate and product time courses in the reaction of [^13^C_6_,D_8_]2DG with yHK

**Figure 5:**
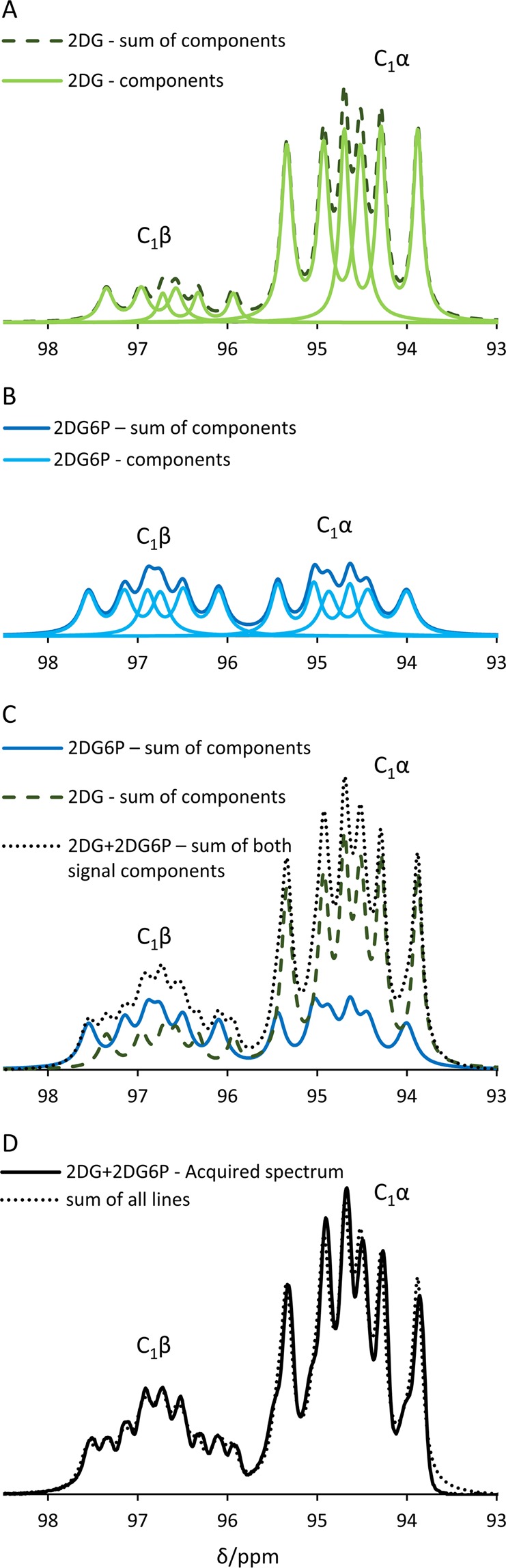
Signal deconvolution of the C_1_β and C_1_α signals to their respective [^13^C_6_,D_8_]2DG and [^13^C_6_,D_8_]2DG_6_P components in one spectrum from the reaction of [^13^C_6_,D_8_]2DG with yHK. This specific spectrum was acquired 14 s after the beginning of the reaction with yHK. The spectrum is taken from the same experiment that is shown in Fig. 2B,A. (**A**) The fitted signal components of C_1_β and C_1_α of [^13^C_6_,D_8_]2DG. The complex signal of each anomer consists of 6 Lorentzian lines due to D-^13^C coupling (J_13C-D_ = 25.5 Hz) and ^13^C-^13^C coupling (J_13C-13C_ = 39.7 Hz). The J-coupling constants reported here are in agreement with those previously reported for [^13^C_6_,D_7_]glucose^50^ and for [1–^13^C]glucose^77^. The ^13^C-^13^C doublet component of each signal was allowed to be asymmetric as asymmetry was previously observed in ^13^C-^13^C split signals in a hyperpolarized state (and occasionally at thermal equilibrium)^38,78,79^. Both fit components are shown in solid light green line, the sum of both components is shown in dashed dark green line. (**B**) The fitted signal components of C_1_β and C_1_α of [^13^C_6_,D_8_]2DG6P. The complex signal of each anomer consists of 6 Lorentzian lines due to D-^13^C coupling (J_13C-D_ = 25.1 Hz) and due to ^13^C-^13^C coupling (J_13C-13C_ = 38.6 Hz). The ^13^C-^13^C doublet component of each signal was allowed to be asymmetric here as well. Both fit components are shown in solid cyan line, the sum of both components is shown in the solid blue line. (**C**) The combination of the 2DG and the 2DG6P fitted signals components. The sum of 2DG fit components is shown in the dashed dark green line, the sum of 2DG6P fit components is shown in the solid blue line, and the sum of both 2DG and 2DG6P fit components is shown in the black dotted line. (**D**) A comparison between the overall fitted signals (black dotted line) shown in (**C**) to the experimental spectrum (solid black line) demonstrates the utility of the deconvolution procedure. The normalized root mean square deviation (NRMSD) between the fit and the experimentally measured spectrum was found to be 9% and 17% for the C_1_β and C_1_α anomers, respectively. 2DG, [^13^C_6_,D_8_]2DG; 2DG6P, [^13^C_6_,D_8_]2DG6P.

To further investigate the underlying reason for the temporal changes in the anomeric ratio in the reaction of [^13^C_6_,D_8_]2DG with yHK we aimed at quantifying the composition of the two anomers in terms of substrate and product during the course of the reaction. Due to the significant overlap between the signals of [^13^C_6_,D_8_]2DG and [^13^C_6_,D_8_]2DG6P in each of the anomer signals, a spectral deconvolution analysis was applied. The C_1_ signals were the only signals where this information could be obtained because: 1) the C_3_-C_5_ signals of [^13^C_6_,D_8_]2DG and [^13^C_6_,D_8_]2DG6P overlapped, 2) in the C_3_-C_6_ signals of both compounds, anomer differentiation was not possible, and 3) as regards to the C_2_ site signals, although this site showed resolved anomer signals, these signals were broader than the signals of the other sites as a result of more extensive splitting due to coupling with two attached deuterons. Consequently, deconvolution of the anomeric signals to their [^13^C_6_,D_8_]2DG and [^13^C_6_,D_8_]2DG6P components was not possible for this site (Supplementary Information S4). An example of the deconvolution operation on the C_1_ signals for one spectrum is shown in Fig. 5.

**Figure 6:**
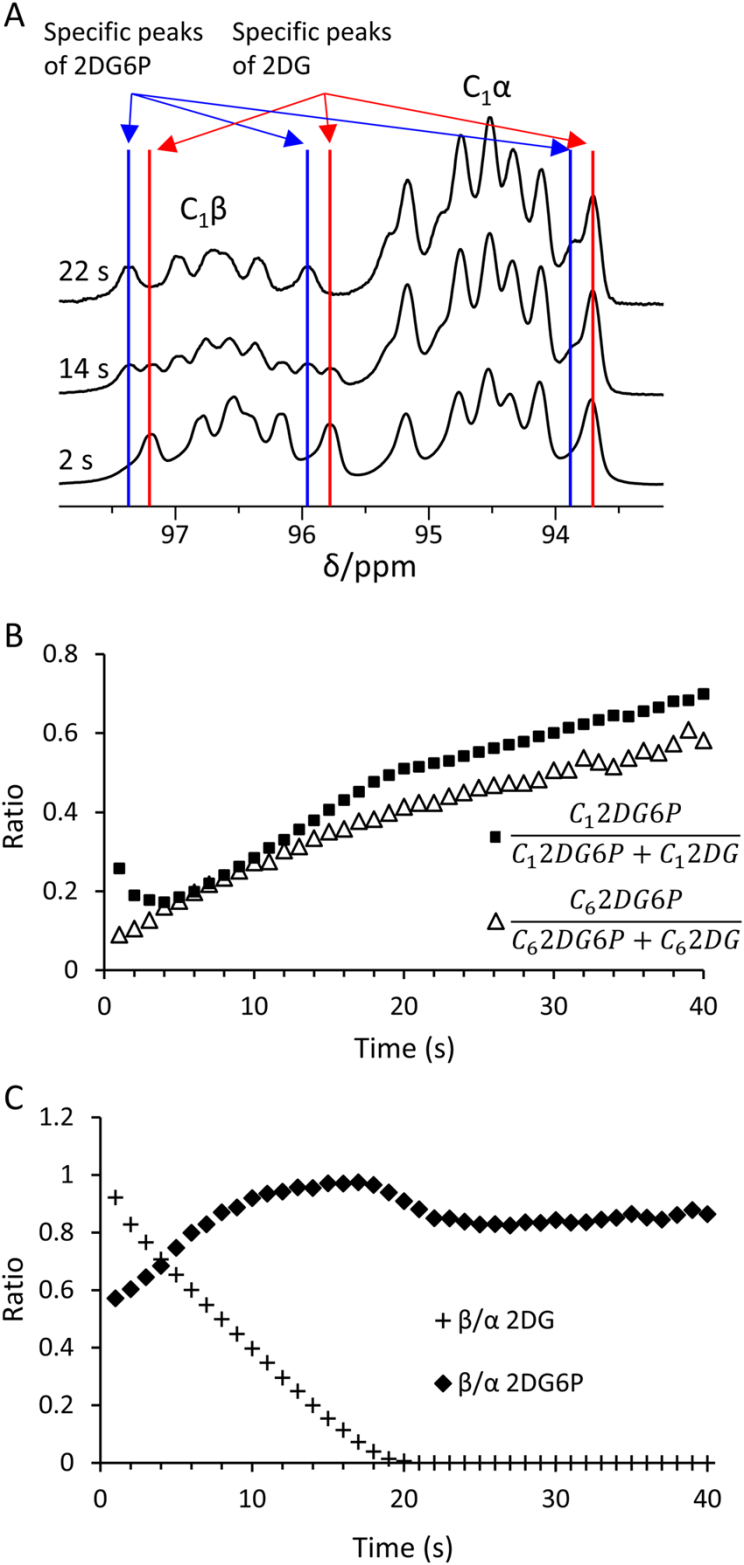
The β/α ratio of [^13^C_6_,D_8_]2DG and [^13^C_6_,D_8_]2DG6P over the course of the reaction with yHK, as calculated using signal deconvolution. (**A**) Spectra acquired at 3 time points from a typical experiment of [^13^C_6_,D_8_]2DG reaction in the presence of yHK (the same experiment is also shown in Figs. 2, 4, and 5). Blue lines indicate the position of signals indicative of [^13^C_6_,D_8_]2DG6P and red lines indicate position of signals indicative of [^13^C_6_,D_8_]2DG. For better visualization of the changes with time in the composition of the specific signals, the spectra at 14 s, and 22 s were multiplied 3-fold and 4-fold relative to the intensity of the spectrum at 2 s, respectively. (**B**) The ratio of product signal to total signal over time for the signals of the C_1_ and C_6_ positions in the same experiment as in (**A**). The rise in this ratio demonstrates the production of [^13^C_6_,D_8_]2DG6P. For the C_6_ position – this conclusion is based on the chemical shift difference of the substrate and the product signals as depicted in Fig. 2B. For the C_1_ position, this conclusion relies on the results of the deconvolution analysis of both anomers. The NRMSD between these two curves was 18%. (**C**) The β/α ratio of the C_1_ signal of [^13^C_6_,D_8_]2DG and [^13^C_6_,D_8_]2DG6P over time in the same experiment.

The ability to deconvolve the complex signals to their respective [^13^C_6_,D_8_]2DG6P and [^13^C_6_,D_8_]2DG components allowed monitoring of the temporal evolution of each of these components in the course of the reaction. The deconvolution procedure relied on the ability to resolve (at least partially) a few unique signals for each of the components (assignment shown in Supplementary Information S5). A typical example of such unique signals is shown in Fig. 6. It can be observed that the [^13^C_6_,D_8_]2DG6P component is increasing with time in both the C_1_α and C_1_β signals (Fig. 6A, blue lines). It can also be seen that the [^13^C_6_,D_8_]2DG component is decreasing in the C_1_β signal in comparison to the C_1_α signal (Fig. 6A, red lines).

The overall proportion of the [^13^C_6_,D_8_]2DG6P component and the [^13^C_6_,D_8_]2DG component, as derived from this deconvolution procedure applied on the C_1_ signals, was in agreement with an analysis of these components in the C_6_ signal, in which the signals of the substrate and the product are resolved, but the anomers are not (Fig. 6B). This result served as a validation for the deconvolution results. The normalized root mean square deviation (NRMSD) for the ratio of product signal to total signal between the C_1_ and C_6_ based determinations was found to be 16 ± 9%, n = 5).

### Anomeric selectivity of yHK for the β anomer of [^13^C_6_,D_8_]2DG

During the reaction with yHK, the β/α ratio of [^13^C_6_,D_8_]2DG (the substrate) appeared decreasing with time, and within no more than 20 s this ratio reached zero as no more β signal was observed (Fig. 6C, plus signs). This finding suggested a selective consumption of the β anomer. On the other hand, the β/α ratio of the product (Fig. 6C, diamonds) appeared increasing throughout the time that the β anomer of the substrate was present, then slightly decreased towards a steady level. Because the anomerization rate of G6P is known to be about 500-fold faster than that of glucose and assuming that the same is true for DG and DG6P, it appears reasonable that this result reflects the fast anomerization rate of the product, *i.e*. [^13^C_6_,D_8_]2DG6P. Since the mutarotation rate of 2-deoxy-D-glucose is more than 10-fold higher than that of D-glucose^58^, these results and the difference from the reaction with [^13^C_6_,D_7_]glucose, cannot be explained by differences in the mutarotation rate of the substrate. Therefore, the results suggest that [^13^C_6_,D_8_]2DG6P is formed in the β anomeric form and then quickly reaches anomeric equilibrium. Thus, in the first part of the reaction, before the consumption of the β anomer of the substrate is completed, we observe two competing processes as regards to the β anomer of the product: on one hand - the production of the β anomer leads to an increase in its signal, and on the other, the continuous rapid establishment of anomeric equilibrium leads to a decrease in this signal. Once the consumption of the β anomer of the substrate is completed, we observe a decrease in the content of the β anomer of the product and arrival to an equilibrium represented by the steady level of this ratio (β/α = 0.92 ± 0.10, n = 5).

## Discussion

2DG in its radioactive form – FDG, is the most abundant tracer in clinical PET examinations. Here, we were interested in researching the ability to monitor the activity of an analog of this tracer in a hyperpolarized state by magnetic resonance. To this end, we have used a 2DG analog uniformly labeled with ^13^C and deuterium as previously done for observing glucose in a hyperpolarized state^38,46–50^. We show here the ^13^C NMR signals of this 2DG analog ([^13^C_6_D_8_]2DG) in a hyperpolarized state with 13% polarization level for the C_1_ site at about 10–11 mM concentration.

After validating that indeed this analog can be detected on single ^13^C NMR acquisitions at this concentration, we utilized it for direct and real-time monitoring of the phosphorylation reactions. In the reaction with yHK, [^13^C_6_,D_8_]2DG was phosphorylated rapidly, but the signal of C_1_β appeared to decay faster compared to the C_1_α signal. This effect was also manifested as a decrease in the β to α ratio of the overlapping C_1_ signal of [^13^C_6_,D_8_]2DG and [^13^C_6_,D_8_]2DG6P during the reaction time. To investigate this effect further, we deconvolved the signal and found that the β/α ratio of the substrate, in particular, decreased until the β anomer was completely consumed. This result suggested that yHK has an anomeric selectivity for the β anomer of [^13^C_6_,D_8_]2DG. The presence of the α anomer of the product is likely explained by a fast anomerization of the newly formed [^13^C_6_,D_8_]2DG6P. This assumption is supported by prior investigations that found that the anomerization rate of glucose-6-phosphate is about 500-fold faster than that of glucose^59^. To the best of our knowledge, the anomerization rate of 2DG6P has not been reported. We note that the lack of anomeric selectivity and the slightly increasing C_1_ β/α ratio seen in the reaction of yHK with hyperpolarized [^13^C_6_,D_7_]glucose are in agreement with the slight anomeric preference of this reaction towards the β anomer observed by Okuda *et al*. in 1984 for hexokinase A in yeast^60^, while Miclet *et al*.^38^ reported comparable kinetics for both anomers of [^13^C_6_,D_7_]glucose.

The yHK used in this study, from the yeast *Saccharomyces cerevisiae*, previously showed similarity to mammalian HKs in terms of nucleotide and amino acid sequence^51^, with 35% amino acid homology compared to the C-terminus end of mammalian hexokinases^53^. Specifically, it was shown that the substrate-binding regions for glucose are conserved between the yHK and the HK found in rapidly growing tumor cells^52^. When comparing the amino acid sequence by global pairwise alignment, we found 29.4% identity and 48.1% similarity between yHK and human HK-2 (Supplementary Information S3). Therefore, this work may represent a preliminary step for studying HK properties of cancerous tissues.

If the anomeric selectivity revealed here for yHK is also present in human HK, the findings of the current study may shed a new light on this important reaction. Various glucose transporters have varying anomeric preferences. For example, intestinal cells^61^ as well as Ehrlich ascites tumors cells^62^ showed preference for the β anomer. In human erythrocytes, initial reports indicated that cross-membrane glucose transport has a preference for the β anomer^63^, but later studies showed no difference between the two anomers^64,65^. In the pancreas, β cells show preferential uptake of β-D-glucose as compared to α-D-glucose and increased insulin release in the presence of α-D-glucose as compared to β-D-glucose^66^. However, this anomeric specificity is perturbed in animal models of diabetes^67,68^. It was also previously shown that the anomeric preference of glucose metabolic enzymes that is observed in normal mammalian cells is absent in tumor cells^69^. If these perturbations are also manifested using [^13^C_6_,D_8_]2DG, it is possible that by imaging the anomeric preference of tissues, cancerous lesions can be detected by such loss of anomeric preference and treatments for diabetes may be monitored as well.

So far, due to the relatively fast anomerization times in physiologic temperature, the investigation of anomeric preference in live tissues was limited. Owing to the high temporal resolution enabled by the dDNP technology, and its translational nature, it may now be possible to re-visit the anomeric preference of enzymatic processes in live tissues and eventually in the whole animal, with the long-term goal of utilizing these differences for differential diagnoses and treatment monitoring.

It is interesting to suggest implications of the anomeric selectivity found here for FDG-PET imaging, which is the most widely used molecular imaging technology for cancer detection and staging. Interestingly, FDG is commonly synthesized from a starting material that is in the β anomeric form^70^. However, it is likely that anomeric equilibrium is established during the synthesis (in the acidification stages^70^) and later on during the time allowed for washout in PET imaging (from injection to imaging, ~1 h). Therefore, it is not clear that standard PET findings should be interpreted any different due to the current findings. However, it may provide useful information for quantitative first-pass PET imaging. In this type of imaging, which is mostly used for research purposes and where the relevant time scales are much faster – on the order of tens of seconds, it is very likely that HK activity is predominantly recorded and it could be useful to consider that the relevant substrate concentration may be only that of the β anomer.

Since its introduction, the only tracer used clinically with hyperpolarized MR was [1-^13^C]pyruvate^32–34^. We believe that the current study may set the foundations for further research regarding imaging possibilities with [^13^C_6_,D_8_]2DG. [^13^C_6_,D_8_]2DG shows beneficial T_1_ profile in the physiological temperature range compared to room temperature with a relative improvement of about 1.6-fold in T_1_ (C_1_ carbon, Supplementary Information S1). Because the short T_1_ relaxation time limits the clinical utility of hyperpolarized ^13^C labeled tracers, this is an important advantage. The current study may also imply that the T_1_ of [^13^C_6_,D_8_]2DG may be further prolonged at higher temperatures and this could enable better preservation of the polarization prior to injection of the hyperpolarized agent to the subject. This aspect will be further investigated in the future.

In the following we will review the pros and cons of a hypothetical MRI scanning with hyperpolarized [^13^C_6_,D_8_]2DG compared to other imaging techniques using a 2DG or glucose tracers.

An important advantage for a dDNP-MRI method over FDG-PET imaging is the ability to detect enzymatic conversion of the imaging probes, *i.e*. that the substrate and product signals can be resolved, and that this detection occurs within seconds. 2DG does not go through further metabolic steps in the glycolysis pathway after its phosphorylation, and is mostly found in its phosphorylated form^71^, 2DG6P, which is intracellular. PET imaging relies on this principle and therefore employs a delay between contrast injection to imaging to allow extracellular FDG to wash out prior to image acquisition. However, with hyperpolarized 2DG there may be no need to wait for the washout of the injected substrate, as the detection of phosphorylated product is possible and occurs in real-time. This short time scale may allow better quantification of enzymatic rates which may provide a new window for tissue characterization and differentiation from normal background tissue activity. These factors may allow imaging of tissues for which the background uptake of FDG is high and limits diagnostic imaging such as the brain and the heart.

Another diagnosis that may benefit from shortening the time from tracer administration to imaging is the characterization of hepatocellular carcinoma (HCC) lesions. The intensity of HCC relative to the normal liver (as in other tissues) in FDG-PET depends on the ratio between the phosphorylation of FDG and the dephosphorylation of FDG-6P by glucose-6-phosphatase^72^. Because glucose-6-phosphatase expression is variable in HCC, FDG-PET imaging only has around 50% sensitivity in HCC detection^73^. It appears possible that shortening the time between the injection of tracer and image acquisition will open new imaging capabilities for HCC as it may enable monitoring of the phosphorylation reaction before the phosphatase reaction takes place.

The major advantage of hyperpolarized MRI as compared to PET imaging is the use of non-ionizing radiation. However, the con of hyperpolarized MRI in this regard is the use of non-trace amounts of the imaging probe. Hyperpolarized [^13^C_6_,D_7_]glucose was visible in 0.12 g/kg in rats^47^, and it is likely that [^13^C_6_,D_8_]2DG will be visible in a similar dose. It is important to note that 2DG was found to be non-toxic in doses up to 0.5 g/kg when administered intravenously to rats^74^. Therefore, it appears likely that [^13^C_6_,D_8_]2DG can be observed in a hyperpolarized state *in vivo* without toxic effects.

Competing MR technologies are gluco-CEST^22,26,27^ and deuterium metabolic imaging (DMI)^29^. The advantage of the gluco-CEST technology is the use of the ^1^H channel that is available on all MRI scanners and the ability to use non-labeled imaging probes (2DG or glucose)^22,26,27^. However, the SNR on gluco-CEST imaging is low. The overall signal change from baseline on CEST imaging was not more than 2-fold^22^, which is far less than that of hyperpolarized MRI as in the latter the background signal is very low. In comparison, the SNR of hyperpolarized [^13^C_6_,D_7_]glucose MRI was 100,000^47^. The advantages of the DMI technology include the ability to use oral administration of the imaging probe and resolve metabolism to lactate, and an improved SNR due to the low background signal^29^. However, the acquisition time of the images was long (*ca*. 30 min)^29^. The con of both gluco-CEST and DMI is the lack of ability to discern the phosphorylated product from the substrate. However, a recent CEST study did show that the signal change observed was higher on DG injection than on glucose injection, suggesting accumulation of DG6P in the brain^22^. Considering these pros and cons of each 2DG/glucose based imaging technology and the central role these imaging probes play in biology and pathology, it is possible that a synergistic combination between them can yield better results than each technique alone.

In summary, we show the potential of [^13^C_6_,D_8_]2DG to serve as an imaging probe for hyperpolarized MRI and find that HK has a unique selectivity for the β anomer of this potential molecular imaging probe, which may provide a new contrast mechanism on MR.

## Methods

### Chemicals

The OXO63 radical (GE Healthcare, UK) was obtained from Oxford Instruments Molecular Biotools (Oxford, UK). [^13^C_6_,D_7_]glucose was obtained from Cambridge Isotope Laboratories (Tewksbury, MA, USA), [^13^C_6_,D_8_]2DG was obtained from 13C Molecular (Fayetteville, NC, USA). Glucokinase from *Bacillus stearothermophilus*, and hexokinase from *Saccharomyces cerevisiae* and all other chemicals used herein were purchased from Sigma-Aldrich (Rehovot, Israel).

### DNP spin polarization and dissolution

Spin polarization and fast dissolution were carried out in a dDNP spin polarization device (HyperSense, Oxford Instruments Molecular Biotools, Oxford, UK) operating at 3.35 T. For polarization, microwave frequency of 94 GHz at 100 mW was applied for 1.4–2.3 h at 1.39–1.45 K. The formulations consisted of 14 mM OXO63 radical and 0.7 or 1.3 mM Gd^3+^, with a 80/100 w/w ratio of 2DG and purified H_2_O, respectively. Prior to insertion to the spin polarizer, the samples were kept at room temperature for *ca*. 2 h to allow for anomeric equilibration of [^13^C_6_,D_7_]glucose and [^13^C_6_,D_8_]2DG. Dissolution was performed with 4 mL of 100 mM TRIS HCl buffer at a pH of 7.6.

### NMR spectroscopy

^13^C NMR spectroscopy was performed in a 5.8 T non-shielded high resolution NMR spectrometer (RS2D, Mundolshein, France) located about 2.2 meters away from the spin-polarization magnet (center-to-center), using a 10 mm broad-band NMR probe. All spectra were acquired with a 10° nutation angle, a repetition time of 1 s, and 19 kHz spectral width.

### Enhancement factor and polarization percent calculation

The enhancement factor was calculated by comparing the maximal integrated signal intensity obtained under hyperpolarized conditions to the integrated signal intensity of the same sample at thermal equilibrium, acquired with the same nutation angle under fully relaxed conditions. In order to compare the integrated intensities from the thermal and hyperpolarized acquisitions, the same acquisition (spectral width, number of points, receiver gain), and processing (exponential line broadening, zero-filling) parameters were used. The thermal equilibrium spectrum was corrected for number of scans. Percent polarization in solution was calculated by multiplying the enhancement factor by the theoretical percent polarization of ^13^C at thermal equilibrium in the same magnetic field (5.8 T).

### Experimental design and enzymatic assays

In experiments using yHK, ATP disodium was dissolved in 1 ml of TRIS HCl medium with MgCl_2_. The pH was then corrected with 10% NaOH solution to 7.6^75^ and ~130 units of hexokinase were added and gently mixed until a clear solution was obtained. This solution was placed in an NMR tube which was placed within the spectrometer’s probe. In experiments with bGK, ATP disodium was dissolved in 2 ml of TRIS HCl medium with MgCl_2_, the pH was corrected and the solution was added into 100 units of bGK. Half of this solution (50 units) was used per experiment. The final reaction concentrations, after combining these enzyme solutions with the dissolution medium, were 20.0 ± 0.5 mM ATP, 10 mM MgCl_2_, and 10.9 ± 0.8 mM of hyperpolarized substrate. The hyperpolarized solution containing [^13^C_6_,D_7_]glucose or [^13^C_6_,D_8_]2DG was injected *via* a Teflon line from the spin-polarizer directly into the NMR tube containing the enzymatic mixture (yHK or bGK) within 3 s of Helium (g) chase. For the experiments shown in Fig. 4D,F, mixing was confirmed by visual inspection for uniformly distributed greenish color indicating adequate mixing of the hyperpolarized substrate before starting data acquisition. For the other experiments, the dissolution of the hyperpolarized substrate was done while the NMR tube was already inside the probe of the NMR spectrometer. The yHK reactions were performed at room temperature (*ca*. 21 °C). The experiments with bGK were performed at 40 °C, achieved by heating both the NMR tube and the dissolution medium transfer line. The temperature of the reactions with bGK was confirmed in real-time by a temperature probe inside the NMR tube (Osensa, Burnaby, BC, Canada).

### Spectral processing and data analysis

Spectral processing was performed using MNova (Mestrelab Research, Santiago de Compostela, Spain). Integrated intensities were calculated either with MNova or with DMFIT^76^. Signal deconvolution analysis was carried out with DMFIT^76^.

### T_1_ calculation

T_1_ determination in hyperpolarized decays measured without enzymatic reactions, and apparent T_1_ determination of hyperpolarized decays measured during enzymatic reactions - not taking into account reaction kinetics, were performed by curve fitting of the signal decay to the following equation $M(t)={M}_{0}\cdot {e}^{(\frac{-t}{{T}_{1}})}\cdot \,\cos \,{\theta }^{(\frac{t}{TR})}$ in which *TR*, the repetition time, and *θ*, the nutation angle of excitation, are known. Curve fitting analysis was performed using Matlab (Mathworks, Natick, MA, USA). In experiments in which enzymatic conversion occurred and for sites in which this reaction could be discerned (the C_6_ position), the T_1_s were determined using a previously published model^39^, which enables the simultaneous determination of both the reaction rate constants and the T_1_s of the substrate and product.

## Supplementary information

Supplementary Information

## Data Availability

The data that support the findings of this study are available from the corresponding author on request.
