# Supplementary material for: [13C6,D8]2-deoxyglucose phosphorylation by hexokinase shows selectivity for the β-anomer
Source: Sci Rep. 2019 Dec 23;9:19683. doi: 10.1038/s41598-019-56063-0 (PMC6928223; doi:10.1038/s41598-019-56063-0)
Supplement: Supplementary file 1 — Supplementary Information [file 41598_2019_56063_MOESM1_ESM.docx]

[^13^C_6_,D_8_]2-deoxyglucose phosphorylation by hexokinase shows selectivity for the β-anomer

Gal Sapir, Talia Harris, Sivaranjan Uppala, Atara Nardi-Schreiber, Jacob Sosna,

J. Moshe Gomori, and Rachel Katz-Brull *

Department of Radiology, Hadassah Medical Center, Hebrew University of Jerusalem, The Faculty of Medicine, Jerusalem, Israel.

Supplementary Information

***S1. The effect of temperature on T_1_ relaxation***

The two enzymatic reactions described in the text required different temperatures. We therefore wished to characterize the T_1_ of the substrates and its dependence on the temperature. In order not to introduce effects of osmolarity or pH, these measurements were conducted in the same medium used for the enzymatic experiments. Table S1 shows that higher temperature (40 °C *vs.* 21 °C) results in longer T_1_ relaxation times. T_1_ times were higher for [^13^C_6_,D_8_]2DG *vs.* [^13^C_6_,D_7_]glucose with T_1_ at 40 °C / T_1_ at 21°C ratios being 1.63 ± 0.04 for [^13^C_6_,D_8_]2DG and 1.37 ± 0.04 for [^13^C_6_, D_7_]glucose.

**Table S1**. The effect of temperature on [^13^C_6_,D_8_]2DG and [^13^C_6_, D_7_]glucose T_1_ relaxation times.

| Compound | T_1_ C_1_β (s) | T_1_ C_1_α (s) | T_1_ C_6_ (s) | T_1_ C_2_β (s) | T_1_ C_2_α (s) |
| --- | --- | --- | --- | --- | --- |
| [^13^C_6_,D_8_]2DG ^a, RT^ | 14.3 | 14.3 | 10.2 | 9.8 | 9.8 |
| [^13^C_6_,D_8_]2DG ^b,^ ^40°C^ | 24.0 ± 1.5 | 23.5 ± 1.4 | 16.7 ± 0.9 | 15.9 ± 0.9 | 15.2 ± 0.3 |
| [^13^C_6_,D_7_]glucose ^a, RT^ | 12.0 | 12.2 | 9.3 | NR | NR |
| [^13^C_6_,D_7_]glucose ^a, 40°C^ | 16.9 | 16.8 | 12.3 | NR | NR |

All measurements were performed in the relevant experimental media described in the Methods, without the presence of enzymes. Only sites that could be spectrally resolved without deconvolution were analyzed. RT, room temperature *ca*. 21 °C; a, n=1; b, n=2; NR, not resolved.

***S2. Reaction kinetics***

The hyperpolarized C_6_ signal time course in the enzymatic experiments was fitted to a kinetic model ^1^ to determine the reaction rate constants. Figure S2 shows a fit to the kinetic model of a typical experiment. The substrate ([^13^C_6_,D_8_]2DG) C_6_ signal is decreasing throughout the experiment, while the product ([^13^C_6_,D_8_]2DG6P) C_6_ signal builds up and then decays. The results of this kinetic model fit to the entire data set described in the text are summarized in Table S2.


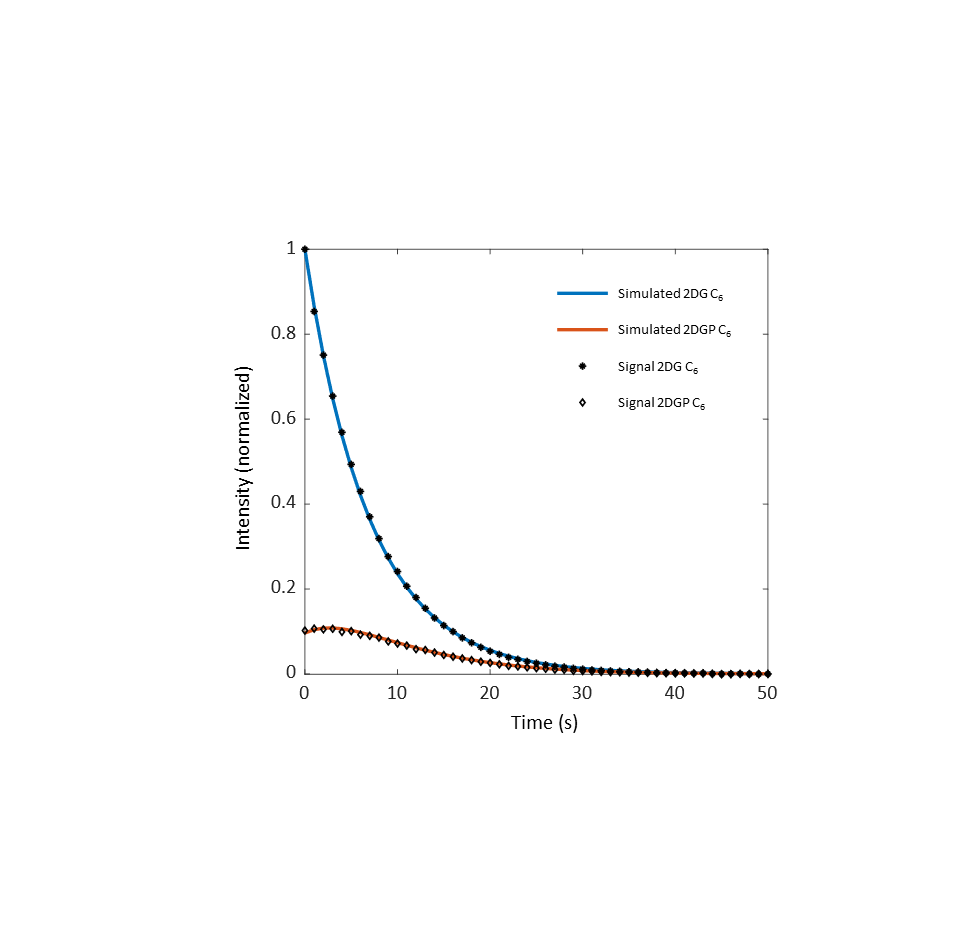


**Figure S2**. Typical time course of the C_6_ signals of [^13^C_6_,D_8_]2DG and [^13^C_6_,D_8_]2DG6P and fitting to a kinetic model.

This plot shows the time course of the normalized signal intensities of the C_6_ carbons of [^13^C_6_,D_8_]2DG (asterisk) and [^13^C_6_,D_8_]2DG6P (diamond) with the simulations created by the kinetic model in solid blue and orange lines, respectively. The R^2^ for the fit between the simulations and the data were 0.9998 and 0.9976 for the C_6_ sites of [^13^C_6_,D_8_]2DG and [^13^C_6_,D_8_]2DG6P, respectively.

**Table S2.** Reaction rate constants and T_1_ values for the C_6_ signals in the phosphorylation reactions.

| Compound & Enzyme | Experiment No. | k (s^-1^) | T_1_ C_6_  sugar* (s) | T_1_ C_6_  sugar-P* (s) |
| --- | --- | --- | --- | --- |
| [^13^C_6_,D_8_]2DG & yHK  RT | 1 | 0.033 | 9.9 | 6.8 |
|  | 2 | 0.027 | 9.8 | 6.5 |
|  | 3 | 0.056 | 9.5 | 7.9 |
|  | 4 | 0.054 | 9.6 | 6.9 |
|  | 5 | 0.050 | 9.5 | 7.7 |
|  | Average ± standard dev. | 0.044 ± 0.013 | 9.7 ± 0.2 | 7.2 ± 0.6 |
| [^13^C_6_,D_7_]glucose & yHK  RT | 1 | 0.022 | 7.9 | 6.1 |
|  | 2 | 0.022 | 8.0 | 6.7 |
|  | 3 | 0.025 | 7.9 | 6.1 |
|  | 4 | 0.026 | 7.9 | 6.5 |
|  | Average ± standard dev. | 0.024 ± 0.002 | 7.9 ± 0.1 | 6.4 ± 0.3 |
| [^13^C_6_,D_7_]glucose & bGK  40°C | 1 | 0.039 | 10.6 | 11.8 |
|  | 2 | 0.037 | 15.2 | 9.8 |
|  | 3 | 0.038 | 13.5 | 9.8 |
|  | 4 | 0.028 | 12.8 | 7.8 |
|  | Average ± standard dev. | 0.035 ± 0.005 | 13.0 ± 1.9 | 9.8 ± 1.6 |

All reactions were performed in the relevant experimental media described in the Methods in the text. RT, room temperature *ca*. 21 °C; sugar, ^13^C and D uniformly labeled 2DG or glucose; sugar-P, 2DG6P or G6P. dev., deviation.

***S3. Homology between yeast and mammalian hexokinases***

yHK shares a 35% amino acid homology compared to the C-terminus end of mammalian hexokinase ^2^. Using EMBOSS (<https://www.ebi.ac.uk/Tools/psa/emboss_needle/>), we compared the amino acid sequence by global pairwise alignment between yeast hexokinase (Uniprot ID G4XSB4) and human hexokinase II (Uniprot ID HXK2). The results of this comparison were as follows: 17.3% identity, 28.3% similarity with 53.0% gaps. When comparing the sequences starting at amino acid #448 for human hexokinase II (including the ATP and glucose binding domains ^3^), we found 29.4% identity, 48.1% similarity and only 23.9% gaps. The lower gap with the higher identity and similarity implicates that the differences in protein chain length account for some of the differences.

***S4. Comparison between C_1_ and C_2_ signals in hyperpolarized [^13^C_6_,D_8_]2DG***

Figure S4 focuses on the signals of the C_1_ and C_2_ sites in a hyperpolarized state, during the reaction with yHK. In both C_2_ signals, and especially in the C_2_β signal, the wider signals which constitute each of the multiplets prevent the deconvolution of the signal to the substrate and the product components. The lower intensity of the C_2_ signals likely results from their shorter T_1_ relaxation times (Table S1).

***
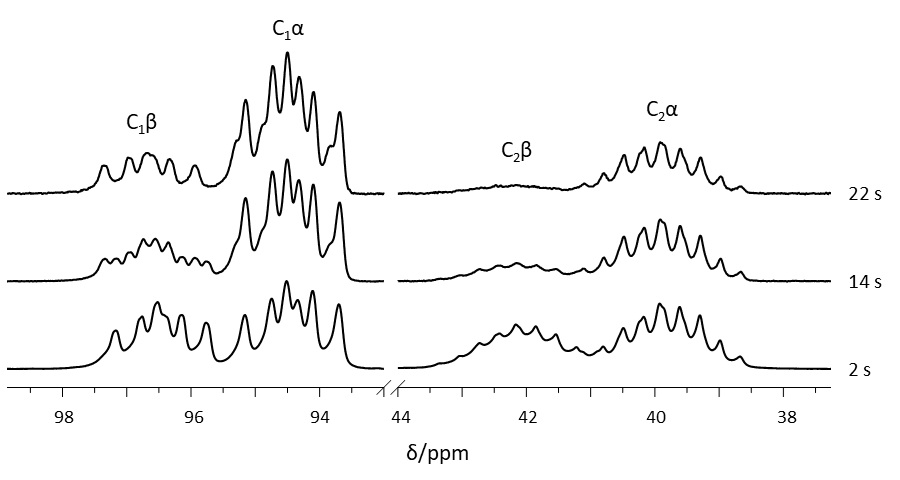
*Figure S4**. The signals of the C_1_ and C_2_ positions of [^13^C_6_,D_8_]2DG at different times points during the reaction with yHK.

The ^13^C spectra shown here were acquired at 3 time points during [^13^C_6_,D_8_]2DG reaction with yHK (the same experiment is shown in Figures 2, 4, 5, and 6). For better visualization, the spectra at 14 s, and 22 s were multiplied 3-fold and 4-fold, respectively, relative to the intensity of the spectrum at 2 s.

***S5. Thermal equilibrium spectra of [^13^C_6_,D_8_]2DG and [^13^C_6_,D_8_]2DG6P and attribution of component specific peaks***

Figure S5 shows the C_1_ spectral region of ^13^C spectra recorded at thermal equilibrium from solutions containing exclusively [^13^C_6_,D_8_]2DG (Figure S5A) or [^13^C_6_,D_8_]2DG6P (Figure S5B). The latter was obtained by combining two of the relevant reaction mixtures that are described in the text (also described in Table S2, line 1 and 2), after the reactions have been completed, lyophilizing them to dryness, and dissolving the resulting powder in 4 mL of 20% D_2_O in water. The former was obtained by the same operation on two reactions mixture containing [^13^C_6_,D_8_]2DG without any enzyme. The spectra are shown on top of the spectra of the reaction in a hyperpolarized state (Figure S5C-E, which are also presented as Figure 6A in the text). The signal peaks that are specific to [^13^C_6_,D_8_]2DG are marked with red lines and those indicative of [^13^C_6_,D_8_]2DG6P are marked in blue lines based on the thermal equilibrium spectra. These unique peaks enabled the deconvolution of these complex signals to their respective substrate and product components, as described in the text.

It can also be appreciated that the two anomers have similar intensities at thermal equilibrium, as would be expected in a solution that had reached anomeric equilibrium. In contrast, in the spectra recorded in a hyperpolarized state, the signals of the β anomer are lower due to the shorter apparent T_1_ times during the reaction with yHK, as described in the text.

***
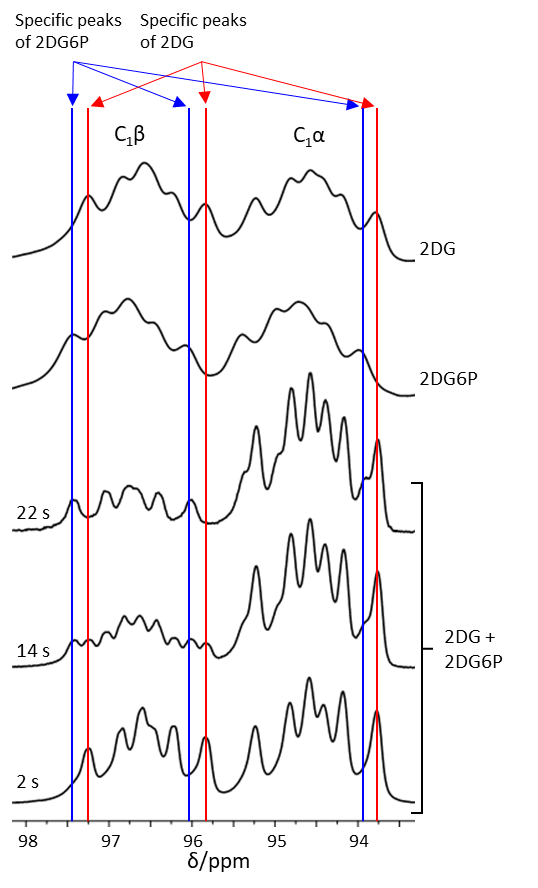
***

A

B

C

D

E

**Figure S5.** ^13^C-NMR spectra of [^13^C_6_,D_8_]2DG and [^13^C_6_,D_8_]2DG6P at thermal equilibrium and during the reaction with yHK in a hyperpolarized state.

**A)** and **B)** Spectra of [^13^C_6_,D_8_]2DG and [^13^C_6_,D_8_]2DG6P, respectively, that were recorded at thermal equilibrium over *ca.* 29 hours each, using 1,320 excitations, 80 s repetition time and 90° flip angle.

**C), D), and E)** These spectra are also shown in Figure 6A in the text. The spectra were acquired during the reaction of yHK with hyperpolarized [^13^C_6_,D_8_]2DG. The spectral processing for all spectra consisted of 10 Hz exponential line broadening with manual phase and baseline correction. The chemical shift scale was referenced to C_6_ of 2DG at 64.0 ppm ^4^ in spectra A, C, D, and E, as described in Figure 1 in the text. Further to this reference, the chemical shift of the C_2_α signal was 40.0 ppm. Spectrum B was referenced to C_2_α signal at 40.0 ppm (in the absence of a C_6_ signal of 2DG in this sample).

2DG, [^13^C_6_,D_8_]2DG; 2DG6P, [^13^C_6_,D_8_]2DG6P.

***Supplementary Note***

***Study Limitations***

The hexokinase enzyme used in this study originates from the yeast *Saccharomyces cerevisiae*. The glucokinase enzyme used in this study originates in *Bacillus stearothermophilus*. We have not checked whether the human equivalents of these enzymes present the same catalytic activity profile. Commercially available human equivalent enzymes appear to have activities that are orders of magnitude lower than the enzymes used in the current study and for this reason were not tested.

**References**

1 Allouche-Arnon, H. *et al.* Quantification of rate constants for successive enzymatic reactions with DNP hyperpolarized MR. *NMR Biomed.* **27**, 656-662, doi:10.1002/nbm.3102 (2014).

2 Miller, S., Ross-Inta, C. & Giulivi, C. Kinetic and proteomic analyses of S-nitrosoglutathione-treated hexokinase A: consequences for cancer energy metabolism. *Amino Acids* **32**, 593-602, doi:10.1007/s00726-006-0424-9 (2007).

3 Nishi, S., Seino, S. & Bell, G. I. Human hexokinase: sequences of amino- and carboxyl-terminal halves are homologous. *Biochem. Biophys. Res. Commun.* **157**, 937-943, doi:10.1016/s0006-291x(88)80964-1 (1988).

4 Navon, G., Lyon, R. C., Kaplan, O. & Cohen, J. S. Monitoring the transport and phosphorylation of 2-deoxy-D-glucose in tumor cells in vivo and in vitro by ^13^C nuclear magnetic resonance spectroscopy. *FEBS Lett.* **247**, 86-90 (1989).
